# Short-Term Axial Length Effects of Combining Paracentral Collimating Apertures with Near-Peripheral Myopic Defocus for Myopia Control

**DOI:** 10.3390/jcm15176856

**Published:** 2026-09-04

**Authors:** Olivia Serdarevic, Edward Yavitz

**Affiliations:** 1 Manhattan Eye, Ear and Throat Hospital, Northwell Health, New York, NY 10065, USA; 2 Kovach Eye Institute, Elmhurst, IL 60126, USA

**Keywords:** myopia, myopia control, axial length, peripheral collimating apertures, paracentral collimating apertures, retinal signaling, peripheral vision, digital, hyperopia, amblyopia

## Abstract

**Background/Objectives**: Myopia is a growing global problem. Current preventative treatments are inadequate, and myopia control spectacles intentionally degrade peripheral optical quality over large retinal areas. Variable results have been related to near-peripheral retinal refractive variability and chromatic refractive spread. A narrow paracentral band of collimating apertures created by annuli with plus-add surrounding appropriately small distance-correcting areas is proposed to decrease defocus and dynamic accommodation cues at the most responsive retinal area for controlling axial elongation while optimizing peripheral vision. **Methods**: Axial lengths (AL), with the contralateral myopic eye serving as the control, were measured in a child with masking after distance and near viewing with a stick-on placed coaxially on a single-vision (SV) spectacle lens during the AL diurnal cycle’s ascending phase only during the experimental sessions at myopia onset and two years later in a case study. The stick-on consisted of a 3.5 mm-wide annular +3D lens containing collimating apertures created by surrounding 1.5 mm-wide circular openings within the stick-on with plus power over the SV minus lens. **Results**: There was no elongation during near viewing in the child’s eye with the stick-on, while the contralateral control eye without the stick-on elongated. The expected increase in elongation during distance viewing during the child’s AL diurnal cycle’s ascending phase was reduced at age 8 and again at age 10 in the eye with the stick-on compared to the eye without the stick-on. Adaptation was immediate. Central and peripheral visual acuities were no different with or without the stick-on. **Conclusions**: This proof-of-concept study in a single child at myopia onset and two years later during myopia progression suggests the potential of this novel strategy for reducing short-term axial elongation during distance and near viewing. Stick-ons, clip-ons, electronic and non-electronic spectacles and contact lenses combining near-peripheral collimation with near-peripheral myopic defocus treatment at the narrow near-peripheral retinal area should be further explored for controlling myopia without reduction of peripheral vision.

## 1. Introduction

The growing myopia epidemic remains a huge global unmet need. The prevalence of this refractive eye condition (nearsightedness) was estimated to be about 2.6 billion in 2020 and is projected to reach about 5 billion by 2050 [1] if effective myopia prevention with widespread global access is not developed. Myopic patients have a 100-fold higher risk of myopic macular degeneration, 3-fold higher risk of retinal detachment, 3-fold higher risk of posterior subcapsular cataract, and an almost doubled risk of open angle glaucoma, with the risk not only increased for high myopia but also for low or moderate myopia [2]. Retinal maculopathy caused by myopia has become a leading cause of irreversible vision impairment and blindness in the middle-aged and the elderly [3].

Myopia is not only becoming more prevalent, but the onset of myopia is occurring increasingly at a younger age. Recent studies of Chinese children have documented that the mean age of myopia onset has been decreasing from 10.6 in 2005 to 7.6 in 2021 [4]. The risk of developing high myopia is greater than 50% if myopia starts before age 8, underscoring the need for effective early intervention to prevent myopia and not only reduce progression of myopia [5]. The likelihood of developing myopia is higher in children with a genetic predisposition and is influenced by various environmental factors [6]. Increased digital screen time, near work, and limited outdoor activities are linked to the recent surge in childhood myopia and decreased age of onset [7]. Children now spend most of their waking hours on near-viewing activities; mean screen time has increased to over 6 h/day for children aged 4–12 [8].

Various pharmacological, light and optical therapies have been developed to slow progression of myopia, but no treatment has yet been shown to prevent myopia [9,10]. Studies with current myopia control therapies have demonstrated variable inter-child efficacy, minimal sustained cumulative reduction of myopia after long-term treatment, and safety concerns, particularly in young children with early onset myopia and high myopia [10,11]. Rebound after cessation of therapy and safety issues have been reported to be greatest after red light therapies and atropine drops applied in the high concentrations needed to produce significant reductions in the elongation of the eye [10,12,13,14,15,16]. Orthokeratology and multifocal contact lenses are not recommended in very young children because of risks of corneal infections and difficult insertion [10]. Optical therapy with spectacles has been considered safer for very young children, but safety concerns have been raised recently regarding long-term use of spectacles with large noncentral areas containing lenslets or cylindrical annular refractive elements or light scattering elements [10,17,18].

It has been suggested that optical therapy intentionally producing peripheral image blur and reduced peripheral contrast sensitivity over large peripheral areas may adversely affect the development of vital visual functions, such as visuomotor coordination and contrast sensitivity, and compromise the brain’s ability to process visual information and additional external noise [10,17,18,19,20,21,22,23,24,25,26]. Different aspects of visual development have different sensitive periods [27]. Development continues until adolescence for many visual functions, such as visual sampling, processing of global motion, coherent motion detection, texture-defined contrast sensitivity, chromatic contrast sensitivity, multisensory integration and visual perceptual learning [28,29,30,31,32,33]. If optical therapy is started at a very young age and continued for over a decade, potential adverse long-term impacts on peripheral visual and motor development from the well-documented degraded peripheral visual inputs may not be known for years. These developmental impacts may only become evident if myopes treated for many years fail to excel in academic, sports, driving or aviation activities [34]. The most recent trends in second-generation spectacles with lenslets increase defocus dioptric power spread over larger areas extending more centrally and peripherally, potentially exacerbating long-term developmental safety risks [35,36]. Prolonged optical treatment from a very early age may be required for myopia prevention and needs to be very safe.

Emmetropization is the controlling process regulating the refractive power of the eye to achieve optimal visual acuity from infancy to adulthood [37]. It is normally a closed-loop feedback system. Stimulatory and inhibitory retinal signaling produce axial lengthening or shortening of the eye by choroidal thinning or thickening, respectively, with diurnal cycle variation and retinal signaling to the sclera for scleral growth modulation [37,38,39,40,41]. Although the cornea and crystalline lens also contribute to the refractive power of the eye and can contribute to myopia causation, axial elongation has been described as the primary factor driving myopia progression [42]. Axial length is the gold standard for measuring the efficacy of myopia control [43]. The complex mechanisms and pathways controlling eye growth are still not completely understood, particularly in very young children [10,44]. Moreover, retinal signaling pathways modulating scleral growth at myopia onset are not all the same as those during myopia progression [44].

Image blur from defocus in the peripheral retina from minus lenses incites retinal signals stimulating axial elongation. Peripheral image blur from plus lenses inhibits axial lengthening at myopia onset but not in all myopic eyes during progression when the feedback system may no longer remain a closed loop [38]. Single-vision (SV) minus lenses for myopia correction at the eye’s center of fixation generally produce off-axis relative peripheral hyperopia because of the shape of the eye. Viewing near objects with distance correction acts as an additional minus lens [37,45].

Importantly, not all peripheral retinal areas contribute equally to signaling from defocus blur. The narrow near-peripheral retinal area between about 6° and 10° eccentricity from the center of fixation (central radius) has been shown recently to be the retinal area most responsive to image blur and defocus sign detection, with little response from the rest of the retina [46,47]. Effective treatment limited to this small near-peripheral retinal area, which has been called the “sweet spot”, may preclude the need to treat large peripheral areas and could decrease the risk of adversely affecting peripheral visual and motor development.

Image blur caused by longitudinal chromatic aberrations (LCA) from off-axis chromatic refractive dispersion-spreads up to 2.5 diopters has been suggested as a primary cue regulating axial growth [38,45,48]. The retina may detect the sign of defocus by comparing defocus in the blue S-cones (which are most dense parafoveally at 9° eccentricity^5^) and the red L- and M-cones [46]. However, some myopic retinas cannot discern defocus in the S-cones, thereby losing the ability to decode the sign of defocus but still responding to near-peripheral defocus with axial elongation, even from plus lenses. Additionally, studies have demonstrated that LCA and parafoveal S-cones, and not monochromatic aberrations, guide accommodation [45,49,50].

Complicating the prevention of excessive eye growth by any optical treatment aimed at preventing relative peripheral hyperopia is the fact that optical errors in separate near-peripheral retinal regions, even at the same distance from the retinal center of fixation, have been shown to be unpredictably variable. Refractive errors differ by up to about 2 diopters of plus or minus power relative to the refractive error at the center of fixation [51]. Peripheral optical errors and axial lengths are different in each child and vary in separate near-peripheral locations in the same child, related to retinal anatomical heterogeneity [51,52,53,54]. Axial lengths vary differently at distinct times of the day and to disparate stimuli at diverse distances from the locus of fixation of the eye [54,55,56]. Therefore, it is not surprising that the efficacy of current myopia control lenses applying the same amount of peripheral myopic defocus in widespread areas throughout the peripheral retina has been reported to vary in children with different peripheral retinal refractions and profiles [52].

A pinhole occluder with a central appropriately small aperture has long been known to collimate light by allowing only parallel rays of light to enter the eye, thereby increasing depth of focus and improving central vision in eyes with refractive errors [57]. We propose using noncentral collimating apertures for myopia control [58]. We hypothesize that a narrow paracentral band of apertures with a diameter small enough to collimate light could reduce defocusing of light in the near-peripheral retina at the “sweet spot” during distance and near viewing, regardless of near-peripheral relative refractive error variability and chromatic spread [58]. Near-peripheral collimating apertures are openings, lenses or other structures limiting the diameter through which only parallel rays are directed to the retina. These collimating apertures could be produced over or within a lens by surrounding appropriately small areas of the lens with an annular area with a different defocus, index of refraction, transparency or other optics. Collimating apertures located paracentrally in or on a lens may eliminate near-peripheral image blur to prevent signals for axial elongation, while also compensating for central and peripheral aberrations. Fixational eye movements produce a moving aperture effect that could increase, in all directions, the retinal areas exposed to collimated light, particularly at the “sweet spot”.

A narrow paracentral band of collimating apertures created by annuli with plus-add surrounding appropriately small distance-correcting areas could be incorporated into stick-ons or clip-ons placed over distance-corrected spectacles or integrated into electronic or non-electronic spectacles or contact lenses. Combining near-peripheral collimation with near-peripheral myopic defocus treatment at the narrow near-peripheral retinal “sweet spot” could be a solution for controlling myopia without exposing the retina to very large areas of degraded images in a developing visual system. Near-peripheral collimating apertures without surrounding opaque areas are desirable to avoid any decrease in retinal illumination or peripheral blind spots. The aperture diameter should provide the best collimation with the least diffraction for optimal visual quality. The prototype that best satisfied those criteria following our investigations of multiple designs contained near-peripheral collimating apertures created by surrounding approximately 1.5 mm-wide areas of the distance prescription with added plus power in a stick-on lens for SV spectacles. This prototype was utilized for studying the feasibility of using near-peripheral collimating apertures created by surrounding distance-correcting paracentral areas with plus-powered annuli to reduce axial elongation while maintaining peripheral visual acuity.

This proof-of-concept case study compared the axial lengths of a young child after short-term near and distance viewing through an SV minus lens in one eye and through a narrow paracentral plus-powered stick-on containing collimating apertures over the SV minus lens in the fellow eye at myopia onset and during myopia progression.

## 2. Materials and Methods

Axial lengths (AL) were measured in a child at myopia onset at age 8 and during myopia progression at age 10, with the fellow myopic eye serving as the control, after distance and near viewing with and without a stick-on consisting of a single, narrow and paracentral +3 diopter (D)-powered annulus containing collimating apertures. The stick-on was worn only during the experimental sessions at age 8 and 10. The stick-on was not worn continuously between age 8 and 10. Informed assent was obtained from the child, and informed consent was obtained from his mother. Approval was obtained from the University of Illinois College of Medicine at Peoria Institutional Review Board. The study was performed in accordance with the tenets of the Declaration of Helsinki.

Non-contact AL measurements were obtained using coherence interferometry (Pentacam AXL Wave (v1.33r05), Oculus, Inc., Arlington, WA, USA). The same experienced technician, masked as to the eye with the stick-on and blinded to prior measurements, obtained three measurements in quick succession at each measurement time (i.e., creating an average of 18 measurements, as the Pentacam measures AL six times/each measurement-capture process and presents the average as the reading). The average of the three readings from the three measurement-capture processes at each measurement time was recorded as the AL, because the standard deviation of the three readings in the child at each measurement time was never greater than 0.004 mm.

All viewings and measurements were performed under photopic conditions (~500 lux) during the morning, because a previous study of AL diurnal cycles in myopic children demonstrated that AL generally rises in the morning and peaks at about 12:30 p.m. [54].

The AL of each eye was measured with spectacles off at 9:00 a.m. after a 10-min “washout” during which the child binocularly viewed cartoons on a television screen at a distance of 30 feet (9.14 m) while sitting with his SV spectacles on to remove effects of any prior near viewing activities or rapid body movements [41].

**Figure 1:**
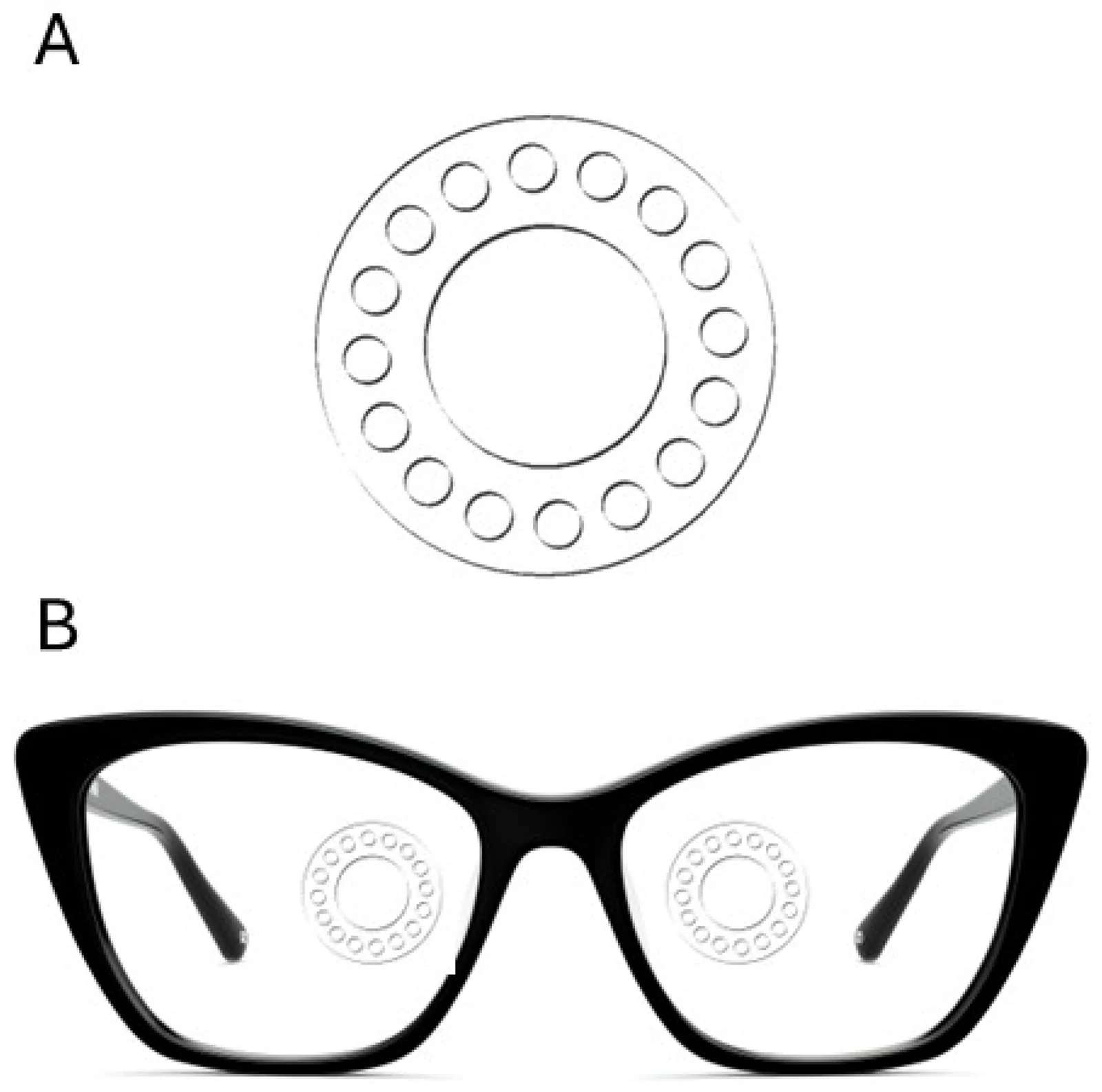
(**A**) Drawing of a transparent polyurethane thermoplastic stick-on lens consisting of a 3.5 mm-wide +3D annular lens with a central 8 mm-wide non-collimating aperture and sixteen 1.5 mm-wide collimating apertures within the annulus. (**B**) Drawing showing the location of the annular stick-on lens on the distance-correcting single vision spectacles’ left lens. When looking through the spectacle lens with the annular stick-on lens centered over the pupil, the left eye is corrected for distance by the −1.50D spectacle lens through the central 8 mm-wide opening of the stick-on lens and in the zone outside the stick-on lens (more than 7.5 m from the optical center). The annular near-peripheral zone of the spectacle lens between 4 mm and 7.5 mm from the optical center is covered by the annular stick-on lens with a power of +3.0D, producing an effective power of +1.5D, except in the areas of the sixteen 1.5 mm-wide collimating apertures that are designed to reduce defocus at the near-peripheral retinal region most responsive to image blur and sign detection.

**Figure 2:**
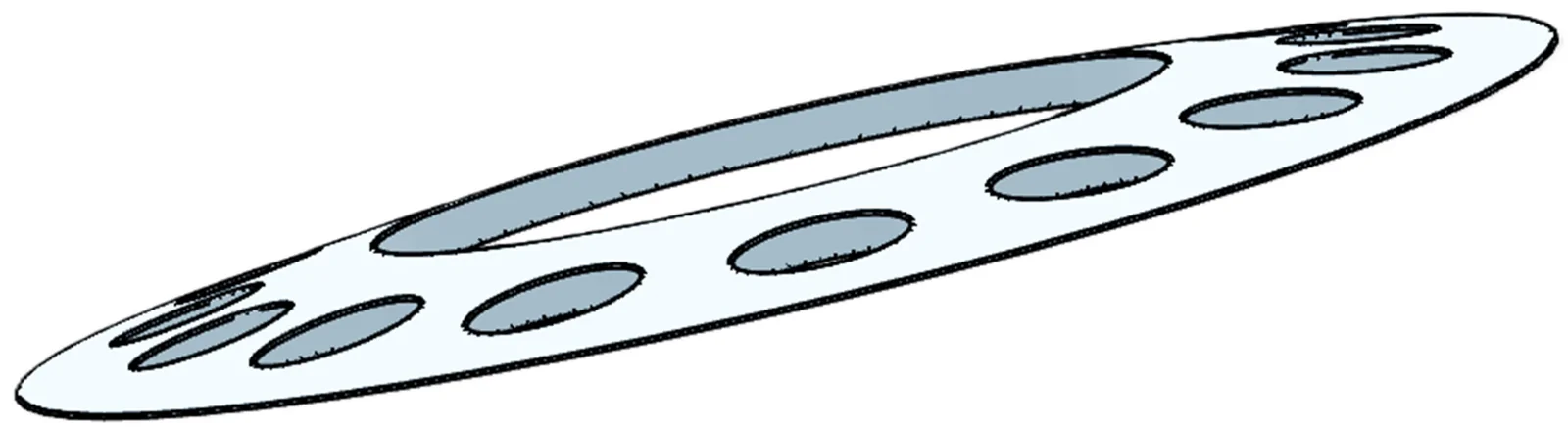
Side view of CAD/CAM model of stick-on lens with collimating apertures.

A transparent polyurethane thermoplastic annular stick-on lens (Proto Labs, Inc., Maple Plain, MN, USA) was then placed onto the left lens of the child’s SV spectacles (Figure 1 and Figure 2), centered over the pupil. The 3.5 mm-wide annular +3D lens had an 8.0 mm-wide central opening and sixteen 1.5 mm-wide openings within the annulus. The 1.5 mm apertures were sufficiently small to collimate light entering the left eye. While looking through the SV spectacle lens with the annular stick-on lens, the left eye was corrected by the SV spectacle minus lens through the central 8.0 mm-wide opening of the stick-on lens and in the zone outside the stick-on lens (beyond 7.5 mm from the optical center). The +3D annular lens produced myopic defocus at the near-peripheral retina but also created collimating apertures by surrounding 1.5 mm-wide circular openings with the +3D lens. The paracentral collimating apertures allowed only parallel light rays to enter the eye [57] and be directed to areas throughout the near-peripheral retina because of fixational eye movements.

The child watched cartoons at a distance of 30 feet for 105 min, after which time each eye’s AL was remeasured. The child then played games looking down at his cell phone held at his preferred near-viewing distance of 14 inches (35.56 cm) for 80 min, after which time each eye’s AL was remeasured.

Ambulatory vision with and without the stick-on was compared, and visual acuities were measured at age 8 and 10 following all AL measurements. Near (14 inches, 35.56 cm) and distance (20 feet, 6.10 m) best-corrected visual acuities (BCDVA, BCNVA) of the left eye were measured with the stick-on lens on the left lens of the child’s SV spectacles (measured when viewing through the central part of the glasses and remeasured when viewing through the annulus of the stick-on) and remeasured after removal of the stick-on lens, which was then discarded.

The increase in axial elongation from age 8 to age 10 due to myopia correction with SV spectacles was calculated. The increase and percentage increase in axial elongation in the left eye with the stick-on lens was compared to that of the contralateral control eye without the stick-on lens after distance viewing during the ascending phase of the diurnal cycle at age 8 and 10. The increase in axial elongation in the eye with the stick-on lens was also compared to that of the contralateral control eye without the stick-on lens after near-viewing at age 8 and 10.

## 3. Results

### 3.1. Study Participant

The Caucasian male studied was diagnosed with myopia at age 8. The child was prescribed SV spectacles (right eye (OD) −0.75D, left eye (OS) −1.50D), because his parents did not elect to have him undergo any myopia treatment other than SV spectacles. His mother had been highly myopic (−8.00D) before undergoing laser vision correction and his father is mildly myopic (−0.75D). There was no history of systemic or ocular abnormalities other than myopia. The child was not on any medications. The child’s mother reported that he spent 7 h/day, on average, performing near viewing activities. Manifest refractions with fogging were OD −0.75D and OS −1.50D. BCDVA and BCNVA were 0.00 logMAR (20/20) in each eye. Mesopic pupil diameters were 5.0 millimeters (mm). Stereopsis, accommodative amplitudes and extraocular movements were normal. Slit lamp and fundus examinations were normal.

### 3.2. Axial Length Measurements and Vision at Age 8

At age 8, ALs measured at 9:00 a.m. were 23.458 mm in the right eye and 23.718 mm in the more myopic left eye. After distance viewing, the ALs were 23.486 mm in the right eye (0.12% increase) and 23.733 mm (0.06% increase) in the left eye. The left eye increased 13 micrometers less than the right control eye. The percentage increase in axial elongation after distance viewing during the ascending phase of the diurnal cycle was reduced by 50% in the eye with the stick-on lens compared to the contralateral control eye without the stick-on lens. After near viewing, AL was 23.733 mm, unchanged, with no axial elongation in the left eye with the stick-on lens, while the contralateral control eye without the stick-on lens lengthened to 23.502 mm, with a 16-micrometer-greater change in AL compared to the left eye. The child adapted immediately to the stick-on lens with no discomfort, dizziness, vertigo, halos, glare or any other subjective difference in central or peripheral vision between the eye with the stick-on lens and the eye without the stick-on lens while either sitting or walking. Objective visual testing demonstrated that BCDVA and BCNVA of the left eye were 0.00 logMAR (20/20) with or without the stick-on lens and while looking peripherally through the annular stick-on.

### 3.3. Axial Length Measurements and Vision at Age 10

Two years after the initial AL measurements, manifest refraction was OD −1.50D, OS −2.50D with BCDVA and BCNVA 0.00 logMAR (20/20) in both eyes and an otherwise normal ophthalmic examination. The child’s mother reported that the child was spending about 9 h/day near viewing and was sleeping about 10 h/night (i.e., spending 64% of waking hours performing near viewing activities at age 10). Axial lengths measured at 9 a.m. after a 10-min washout period were OD 23.959 mm and OS 24.477 mm, increasing by 0.501 mm in the right eye and 0.759 mm in the left eye between age 8 and 10 and confirming the diagnosis of progressive myopia [59]. After distance viewing for 105 min, AL was 23.979 mm (0.08% increase) in the right eye and 24.484 mm (0.03% increase) in the left eye. The left eye increased 13 micrometers less than the right eye. The percentage increase in axial elongation after distance viewing during the ascending phase of the diurnal cycle was reduced by 63% in the eye with the stick-on lens compared to the contralateral eye without the stick-on lens. After near viewing for 80 min, there was no axial elongation in the left eye with the stick-on lens, while the contralateral control eye without the stick-on lens lengthened with a 15 micrometer-greater change in AL compared to the left eye. The child adapted immediately to the stick-on lens with no discomfort or subjective difference in central or peripheral vision between the eye with the stick-on lens and the eye without the stick-on lens while either sitting or walking. BCDVA and BCNVA were 0.00 logMAR (20/20) with or without the stick-on lens and through the annulus of the stick-on.

## 4. Discussion

This proof-of-concept study in a single child suggests the feasibility of reducing short-term axial elongation without reducing peripheral visual acuity by placing a narrow annular plus-powered stick-on lens containing collimating apertures over the paracentral region of an SV distance-correcting spectacle lens. There was no elongation during near viewing in the child’s eye with the stick-on, while the contralateral control eye without the stick-on elongated. The expected increase in elongation during distance viewing during the child’s AL diurnal cycle’s ascending phase was reduced at myopia onset at age 8 and again during myopia progression at age 10 in the eye with the stick-on compared to the eye without the stick-on. Stone et al. recently concluded that there are “important mechanistic differences between the onset of myopia and the progression of established myopia” and “clinical investigations should systematically incorporate circadian mechanisms… in seeking more effective treatments to normalize eye growth in children.” [44]. Before this case study, short-term, few-hour changes in AL had been used to study myopia control spectacles with plus power added to distance correction over very large peripheral retinal areas in young adults in whom retinal signaling at the “sweet spot” is not identical to that in young children [41,44,60]. This case also provides distinctive findings because those prior studies did not compare SV lenses with SV lenses having noncentral added plus lenses during the same portion of the circadian ascending or descending phase in all eyes and did not compare AL responses both at myopia onset and during myopia progression [41,60].

The child in our case study had a genetic predisposition and was spending most of his waking hours near viewing. Short-term axial lengthening has been reported to occur in children with and without myopia after viewing accommodative stimuli simulating near viewing, with more lengthening observed in myopic eyes, the amount of which was consistent with the increases in AL in the myopic eye without the stick-on lens in our study [56]. Hughes et al. suggested that this axial elongation during near viewing “may result in longer-term eye growth because of summation of accommodation-induced eye elongation, with the eyes of myopic children potentially being more susceptible.” [56]. Given the recent striking increase in near viewing by children, prevention of axial elongation during near viewing may further improve myopia control. It has been hypothesized recently that increased accommodative pupil constriction from negative lenses reduces retinal illumination, weakening retinal responses and contributing to axial elongation [61].

Current peripheral defocus-based spectacles for myopia control with lenslets or cylindrical annular refractive elements do not decrease dynamic accommodative responses [62]. According to Charman et al., images produced by the SV lens in the central optical zone, and not the lenslets, drive accommodative responses, because “the lateral separation between the in-focus images produced by different lenslets results in a confused image” [25]. In our case study, there was no short-term axial elongation during near viewing with the narrow paracentral +3D annular stick-on containing collimating apertures, whereas, at the same time of the AL diurnal cycle, the control SV lens alone caused elongation. Central pinholes have been documented to produce 3 diopters of depth of focus for a visual acuity of 20/50, which is equivalent to 5 cyc/deg, the visual acuity in the parafoveal S-cone system [46,57]. The paracentral stick-on with collimating apertures, which were created by surrounding 1.5 mm-wide circular openings over the SV minus lens with added plus power, may have increased the defocus range up to about 3 diopters at the near-peripheral retina. We hypothesize that this large depth of focus may have neutralized and compensated for both monochromatic and polychromatic optical aberrations, including longitudinal chromatic aberrations that have been shown to drive signals for dynamic accommodation and retinal elongation [50]. Future studies will need to confirm whether near-peripheral collimating apertures surrounded and created by added plus power reduce near-peripheral retinal accommodation and defocus cues.

Previous studies have shown that some myopic retinas lose the ability to decode the sign of defocus, leading to elongation from both myopic and hyperopic defocus [38]. However, even if plus lenses were no longer able to trigger the inhibitory arm of the emmetropization feedback loop, the near-peripheral collimating apertures may be able to increase depth of focus throughout the most responsive retinal “sweet spot” and remove image blur cues that could trigger axial elongation. It is also becoming increasingly evident that effective treatment to prevent myopia, or at least greatly control abnormal axial elongation as soon as possible, must be developed to prevent ongoing molecular and metabolic reprogramming and physiological stress causing increasing neural strain and dysfunction. Progressive myopia first affects inner retinal circuitry with synaptic dysregulation and disrupted retinal signaling [63]. Progressive myopia, if very rapid or very prolonged, also causes retinal photoreceptor dysfunction and widespread retinal structural abnormalities [63]. A disposable plus-powered stick-on lens containing paracentral collimating apertures should be further explored for providing convenient and safe myopia control not only after the onset of myopia but also, very importantly, during pre-myopia in very young children. In the future, near-peripheral collimating apertures may be used synergistically, if necessary, with neuromodulating or scleral collagen-strengthening pharmacologic therapies or light treatments or refractive surgery.

Studies have objectively quantified, by several measures, the poor optical quality, image degradation and reduced image contrast produced over large retinal areas by myopia control spectacles with lenslets or cylindrical annular refractive elements or light scattering elements [18,19,20,21,22,23,24,25,26]. Ding et al. showed that contrast sensitivities (areas under the log CSF) were significantly lower in children wearing lenses with highly aspherical lenslets for at least 6 months than in the SV group under both zero-noise conditions and high-noise conditions, despite similar intrinsic noise levels of the visual system of the two groups [18]. Notably, the perceptual template gain (β), which is a measure of how efficiently the visual system processes contrast information to exclude external noise and detect stimuli, was significantly lower for low spatial frequency stimuli (2 cycles per degree) in the children wearing lenses with lenslets for at least 6 months [18]. Children wearing currently available myopia control spectacles have been documented to adapt to the induced peripheral blur variably over time [19]. However, this neural adaptation may impair activities requiring fine spatial discrimination. Barbot et al. concluded that prolonged exposure to optically degraded retinal inputs results in neural insensitivity to fine spatial details [64]. Measurements of static standard visual fields or microperimetry retinal sensitivity average thresholds have not been performed in very young children. Even in older children, these measurements are too coarse and too unreliable, with high fixation losses and high test–retest variability, to evaluate the safety of degraded peripheral visual quality for dynamic visual processing in developing visual systems [35,65]. A narrow paracentral area containing appropriately sized collimating apertures created by surrounding added plus power has the potential to reduce axial elongation without producing adverse retinal image alteration over large retinal areas, maintain (and maybe even improve) immediate and long-term peripheral visual acuity and motion detection, and compensate for any progressive increase in myopia or astigmatism between changes in spectacles.

This first report of a narrow paracentral band of plus add incorporating collimating apertures provided proof of concept and insights into AL findings during short-term near and distance viewing during the AL diurnal cycle’s ascending phase in a young child at the onset of myopia and during myopia progression. Study strengths included AL measurements with masking and blinding at myopia onset and during progression two years later. Due to the known short-term diurnal variability of AL measurements, the contralateral myopic eye serving as the control was another strength, ensuring that AL measurements were made during the same portion of the diurnal curve in both eyes. The left treated eye was more myopic than the right control eye at baseline at myopia onset at age 8 (−1.50D vs. −0.75D) and at baseline during myopia progression at age 10 (−2.50D vs. −1.50D). Our study purposely used the more myopic left eye as the treated eye, because children’s eyes with higher myopia have been shown to be more responsive with axial elongation to accommodative signals than less myopic and emmetropic eyes [56]. If the stick-on had not been used, the axial length of our child’s more myopic left eye would have been expected to increase more than his less myopic right eye during near viewing. Therefore, the AL results with the stick-on on the more myopic eye may have even underestimated the ability of the stick-on to reduce axial elongation. Axial elongation during distance viewing starting shortly after 9:00 a.m. was during the same portion of the ascending phase of the diurnal curve in both eyes and was reduced in the eye with the stick-on. The axial elongation observed only in the control eye without the stick-on during near viewing after 11:00 a.m. was during the flatter part of the diurnal curve near the peak in both eyes and, therefore, probably predominantly caused by the near stimulus. The standard deviation of the three measurement readings at each measurement time was never greater than 0.004 mm (margin of error ±0.00045 at 95% confidence level). The experience of the technician and the excellent cooperation of the child during quickly repeated measurements, as well as the absence of high myopia, astigmatism, cataracts or other ocular media opacities, contributed to the consistency of the AL measurements. The standard deviations of our child’s AL measurements were consistent with the within-subject standard deviations in AL studies in cooperative young adults with little to no astigmatism and no ocular media opacity [66].

Our single-participant study avoided confounding variables inherent in some previous studies measuring AL over a few hours in multiple participants, such as standard deviation of a total group’s AL being more than 100× greater than individual AL changes over the few-hour study; myopes at different stages of progression (some at onset, some progressing and some stabilized); unnatural monocular viewing; and AL diurnal cycle ascending and descending phases not accurately determined and differing in timing and amplitude in different subjects and during sequential measurements [60]. For these reasons, as well as the difficulty of reliably performing a multi-hour study in young children (who may not be as mature or complete task performance and AL measurements as well or with as little movement as our participant), performing this same study in a large group of children probably would not be as scientifically valuable. Despite the valuable findings from our case study, the single participant limits the generalizability of some of this study’s findings. Further explorations of near-peripheral collimating apertures should include modeling with ray tracing and image analysis to determine lens designs for optimal through-focus image quality and depth of focus. Following determination of optimal lens designs in stick-ons, clip-ons or spectacles, investigations of multiple visual parameters through the central and peripheral zones, accommodation with pupil size measurements, and long-term axial length should be investigated in large groups of children. Prospective multicenter randomized clinical trials with collimating apertures created by added plus power should then be designed to avoid the limitations of many myopia control studies assessing multiyear efficacy [67]. Additionally, near-peripheral collimating apertures created by surrounding added minus power could be explored for control of hyperopia, which also has been associated with serious ocular diseases [68].

Future investigations should also include liquid crystal glasses designed to create paracentral collimating apertures at high speeds for better functionality and configured for calibration, customization and monitoring. Digital treatment with near-peripheral collimating apertures may provide temporal modulation controllable by the parent or remotely by the treating physician based on the child’s activities, the child’s diurnal timing of elongation “go” signals, treatment response or adherence. Temporal modulation of eye exposure to near-peripheral collimating apertures at speeds between 100 hertz and 300 hertz may be customized based on a child’s individual speed of neural processing of visual information for potential simultaneous integration before perception by the child. High-speed temporal modulation of collimating apertures also might be able to increase visual resolution beyond retinal photoreceptor limits by improving cortical temporal and spatial neural integration. Blurred edges have been demonstrated to look up to 50% sharper when they are presented briefly (8–24 ms) than at longer durations (100–500 ms), even without motion and even at low contrast [69]. Electronic glasses could also be designed to provide control of myopia or hyperopia with simultaneous treatment of amblyopia by combining near-peripheral collimating apertures with alternating decreased contrast or occlusion of the central optical zone in each eye. Such combined digital treatment should be explored for peripheral binocular stimulation and fusion, as amblyopia has been recognized as a binocular central visual problem with abnormal fixation but with relative sparing of peripheral vision [70,71,72,73].

## 5. Conclusions

A paracentral narrow band of collimating apertures can be created by annuli with plus add surrounding appropriately small distance-correcting areas in a stick-on or clip-on lens for SV spectacles or incorporated directly into electronic or non-electronic spectacles or contact lenses. Near-peripheral collimating apertures are proposed for increasing depth of focus and reducing defocus at the near-peripheral retinal signaling “sweet spot” to prevent axial elongation during near viewing and overcome variable near-peripheral defocus signals related to retinal refractive variability and chromatic refractive spread. This proof-of-concept study in a single child at myopia onset and two years later during myopia progression suggests that placing a narrow paracentral plus-powered stick-on with collimating apertures over distance-correcting spectacles can reduce short-term axial elongation. Larger controlled studies demonstrating longer-term axial length effects and sustained myopia control in children wearing lenses with paracentral collimating apertures are required before any clinical recommendation can be made. Combining near-peripheral collimation with near-peripheral myopic defocus treatment should be further explored for controlling myopia while optimizing peripheral visual and motor development.

## 6. Patents

Aperture in Motion, LLC (Phoenix, AZ, USA), is the sole owner and assignee of US Patent No. 10386646, US Patent No. 11106043, US Patent No. 11480798, US Patent No. 11982815 and US Patent No. 12566331.

## Data Availability

The data generated during this study are available from the corresponding author on reasonable request.
